# A Practical Approach to Polycythemia in the Outpatient Setting and Its Importance

**DOI:** 10.7759/cureus.19368

**Published:** 2021-11-08

**Authors:** Helda Souresho, Michael Mgerian, Suzanne Havican, Elizabeth Suniega, Carol Gambrill

**Affiliations:** 1 Family Medicine, Graduate Medical Education, Hospital Corporation of America (HCA) Houston Healthcare West, Houston, USA

**Keywords:** embolic events, ischemic cva, myocardial infarction, polycythemia vera, erythrocytosis, polycythemia

## Abstract

Polycythemia left undiagnosed or untreated may result in a number of sequelae including myocardial infarction or cerebral vascular accidents. While the diagnostic criteria, classification, and workup are established, many practitioners fail to either initiate the process or perform the correct workup. Most clinicians are familiar with polycythemia and its respective clinical encounters, nevertheless, the fact that it is so frequently misdiagnosed or improperly worked up necessitates additional education. This case report covers three practical clinical examples of outpatient polycythemia, and their respective workups Furthermore, this publication will discuss the diagnostic criteria laid out by the World Health Organization and the confusion regards complications based on etiology.

## Introduction

Borderline-to-mild elevations of hemoglobin, or hematocrit, are a common issue encountered in the outpatient primary care setting that is often undiagnosed, incompletely, or inappropriately worked up. While polycythemia and its sequelae are well studied, they are often underdiagnosed by primary care clinicians due to unfamiliarity with revised diagnostic and testing guidelines [1].

The prevalence of polycythemia vera (PV) in the United States is 44-57 per 100,000 (0.044-0.057%) [1], while the prevalence of erythrocytosis (polycythemia) was 0.3% by the strict (old, 2008) criteria and 3.4% by wide (new, 2016) criteria [2,3]. Since the new criteria have been implemented, there have been many questions regarding complications in patients with erythrocytosis regardless of the etiology and if the risk of thrombosis is the same in both. 

In this case report, we present three cases encountered in a primary care setting. While the individual cases are not exceptionally rare, they are examples of common encounters and the appropriate workup for each case. This study will also review erythrocytosis differentials, consequences, and the current World Health Organization (WHO) guidelines and recommendations for the workup of polycythemia vera. This study aimed to bring together a practical clinical perspective on polycythemia, the evidence-based methodology for its appropriate workup, and to acknowledge the consequence of misdiagnosis.

## Case presentation

Case 1

A 47-year-old Middle Eastern male with a past medical history of dyslipidemia and obesity with a BMI of 30 presented to the clinic for concerns about memory loss and sleeping problems. During the initial visit, the patient endorsed symptoms of difficulty staying asleep and several nocturnal awakenings per night with urges to binge eat. According to the patient's partner, he has been snoring and often gasping for air while sleeping for years. He has been having difficulties learning the English language due to poor memory, which has become very frustrating. He admitted to having sleeping problems, fatigue, loss of concentration, depressed mood, and loss of interest in doing activities he previously enjoyed but denied any suicidal or homicidal ideation. His depression screening was consistent with moderate depression. Mini-mental exam score yielded 23/30 which was suggestive of mild dementia.

On physical examination, he was afebrile, normotensive, heart rate (HR) was 71 beats per minute, respiratory rate (RR) was 18 breaths per minute, oxygen saturation was 98% on room air, and blood pressure (BP) was 117/82 mmHg. Physical examination was unremarkable other than mild scattered wheezes across all lung fields. On a mental status exam (MSE), the patient had constricted but appropriate affect, mild psychomotor retardation; otherwise normal MSE. 

The patient was started on duloxetine for depression. Workup revealed absolute erythrocytosis due to secondary causes. The patient was seen two weeks later, he reported no changes in his symptoms. Follow-up labs were ordered to workup his erythrocytosis (Table 1). Further recommendations for a sleep study, initiating 81 mg aspirin, smoking cessation, and nicotine replacement were recommended. Regular follow-ups for hemoglobin/hematocrit (Hb/Hct) checks were discussed to ensure the prevention of thrombotic events. 

**Table 1:** Laboratory results of case 1 ANA: antinuclear antibody; TSH: thyroid-stimulating hormone; TIBC: total iron-binding capacity; ESR: erythrocyte sedimentation rate; EPO: erythropoietin; RPR: rapid plasma reagin

Lab Results: Initial Encounter		
Test Name	Results	Reference Range
Hemoglobin	18.1	Males: 13.2-16.5 (g/dL)
		Females: 11.6-15.0 (g/dL)
Hematocrit	50.7	Male: 38.3-48.6 (%)
		Female: 35.5-44.9 (%)
Platelets	204	150-450 (×10^3^/uL)
ANA Direct	Negative	Negative
TSH	1.54	0.450-4.500 (uIU/mL)
Lab Results: Subsequent Encounter		
Hemoglobin	17.7	Males: 13.2-16.5 (g/dL)
		Females: 11.6-15.0 (g/dL)
Hematocrit	50.8	Male: 38.3-48.6 (%)
		Female: 35.5-44.9 (%)
Iron	118	38-169 (ug/dL)
Iron Saturation	36	15-55 (%)
TIBC	330	250-45 (ug/dL)
Ferritin	122	30-400 (ng/dL)
Transferrin	274	177-329 (mg/dL)
Urine Studies	Normal	Normal
Testosterone	410	264-916 (ng/dL)
ESR	2	0-15 (mm/h)
EPO	7.3	2.6-18.5 (mLU/mL)
JAK2V617F	Negative	Negative
Lab Results: Initial Encounter		
Hemoglobin	20.3	Males: 13.2-16.5 (g/dL)
		Female: 11.6-15.0 (g/dL)
Hematocrit	62.0	Males: 38.3-48.6 (%)
		Females: 35.5-44.9 (%)
Platelets	137	150-450 (×10^3^/uL)
Lab Results: Subsequent Encounter		
Hemoglobin	15.6	Males: 13.2-16.5 (g/dL)
		Females: 11.6-15.0 (g/dL)
Hematocrit	46.1	Males: 38.3-48.6 (%)
		Females: 35.5-44.9 (%)
ANA Direct	Negative	Negative
RPR	Non-Reactive	0.450-4.500 (uIU/mL)
ESR	7	0-15 (mm/h)
EPO	5.2	2.6-18.5 (mLU/mL)
JAK2V617F	Negative	Negative

Case 2

A 45-year-old male with a history of tetralogy of Fallot (status post-surgical correction in childhood) presented for an annual physical examination after recently migrating from Iraq; the patient had no acute complaints or symptoms to report. On physical examination, the patient was afebrile, HR was 61 beats per minute, BP was 109/71 breaths per minute, and his BMI was 25.4. The patient is a non-smoker and has a negative screen for sleep apnea. Medical labs from that encounter are shown in Table 2.

**Table 2:** Laboratory results of case 2 ANA: antinuclear antibody; ESR: erythrocyte sedimentation rate; EPO: erythropoietin; RPR: rapid plasma reagin

Lab Results: Initial Encounter		
Test Name	Results	Reference Range
Hemoglobin	20.3	Males: 13.2-16.5 (g/dL)
		Females: 11.6-15.0 (g/dL)
Hematocrit	62.0	Males: 38.3-48.6 (%)
		Females: 35.5-44.9 (%)
Platelets	137	150-450 (×10^3^/uL)
Lab Results: Subsequent Encounter		
Hemoglobin	15.6	Males: 13.2-16.5 (g/dL)
		Females: 11.6-15.0 (g/dL)
Hematocrit	46.1	Males: 38.3-48.6 (%)
		Females: 35.5-44.9 (%)
ANA Direct	Negative	Negative
RPR	Non-Reactive	0.450-4.500 (uIU/mL)
ESR	7	0-15 (mm/h)
EPO	5.2	2.6-18.5 (mLU/mL)
JAK2V617F	Negative	Negative

The patient was notified of his significantly elevated levels of hemoglobin (20.3) and was asked to return to repeat his complete blood count (CBC) and subsequent labs; however, he was lost to follow-up. In the interim period of his loss to follow-up, the patient was seen by cardiology following an emergency department visit for dizziness and shortness of breath. During his follow-up with cardiology, a transthoracic echocardiogram revealed left to right shunting suggestive of Eisenmenger syndrome secondary to his previously treated tetralogy of Fallot (ToF). The patient underwent cardiac surgery for ToF with an effective resolution of his signs and symptoms. 

On his nine-month post-operative follow-up, the patient's labs revealed normal Hb/Hct; his erythropoietin serum sample was lost and therefore the test was not performed. Given the patient's otherwise negative polycythemia workup and known history of ToF with recurrence of Eisenmenger syndrome, no further workup was indicated.

Case 3 

A 59-year-old male, a recent immigrant from Malaysia, presented to the clinic for an annual physical examination. He had a history of “thyroid disorder” and hypertension, for which he was taking lisinopril and methimazole, which he had been taking for over 10 years. The patient endorses complaints of intermittent brief episodes of palpitations and periodic facial flushing, which have been present for a week or so; he had no other complaints. On physical examination, the patient was found to have mild blanching erythema over his anterior neck and face; the remainder of his examination was unremarkable. The patient’s labs are referenced in Table 3.

**Table 3:** Laboratory results of case 3 TSH: thyroid-stimulating hormone; TPO Ab: autoantibodies to thyroid peroxidase

Lab Results: Initial Encounter		
Test Name	Results	Reference Range
Hemoglobin	16.4	Males: 13.2-16.5 (g/dL)
		Females: 11.6-15.0 (g/dL)
Hematocrit	52.1	Males: 38.3-48.6 (%)
		Females: 35.5-44.9 (%)
Platelets	312	150-450 (×10^3^/uL)
TSH	<0.005	0.450-4.500 (uIU/mL)
T4, Total	10.0	4.5-12.0 (ng/dL)
T4, Free	4.16	0.82-1.77 (ug/dL)
TPO Ab	>600	0-34 (Iu/mL)

Although this patient's hemoglobin is below the WHO diagnostic and workup criteria for polycythemia vera, his hematocrit is elevated. He was sent for a thyroid uptake study, his hyperthyroid medication was adjusted and was subsequently advised to follow-up for repeat labs to reassess his thyroid function and hematocrit. At the time of the encounter, or after review of initial labs, erythropoietin levels and JAK2 mutation were not ordered.

## Discussion

**Figure 1:**
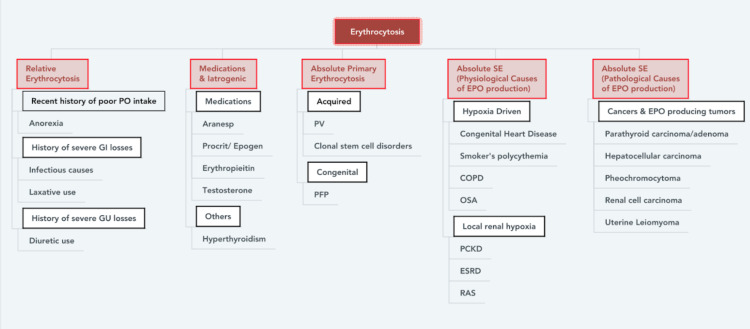
Erythrocytosis classification SE: secondary erythrocytosis; PV: polycythemia vera; PFP: primary familial polycythemia; COPD: chronic obstructive pulmonary disorder; OSA: obstructive sleep apnea; PCKD: polycystic kidney disease; ESRD: end-stage renal disease; RAS: renal artery stenosis

Erythrocytosis (alternatively called polycythemia) is characterized by an increase in the production of red blood cells. It is critical to recognize how these laboratory findings may lead to a wide range of differential diagnoses and their etiology (Figure 1). To arrive at the final diagnosis, a comprehensive medical history and an appropriate diagnostic workup are indicated. 

**Figure 2:**
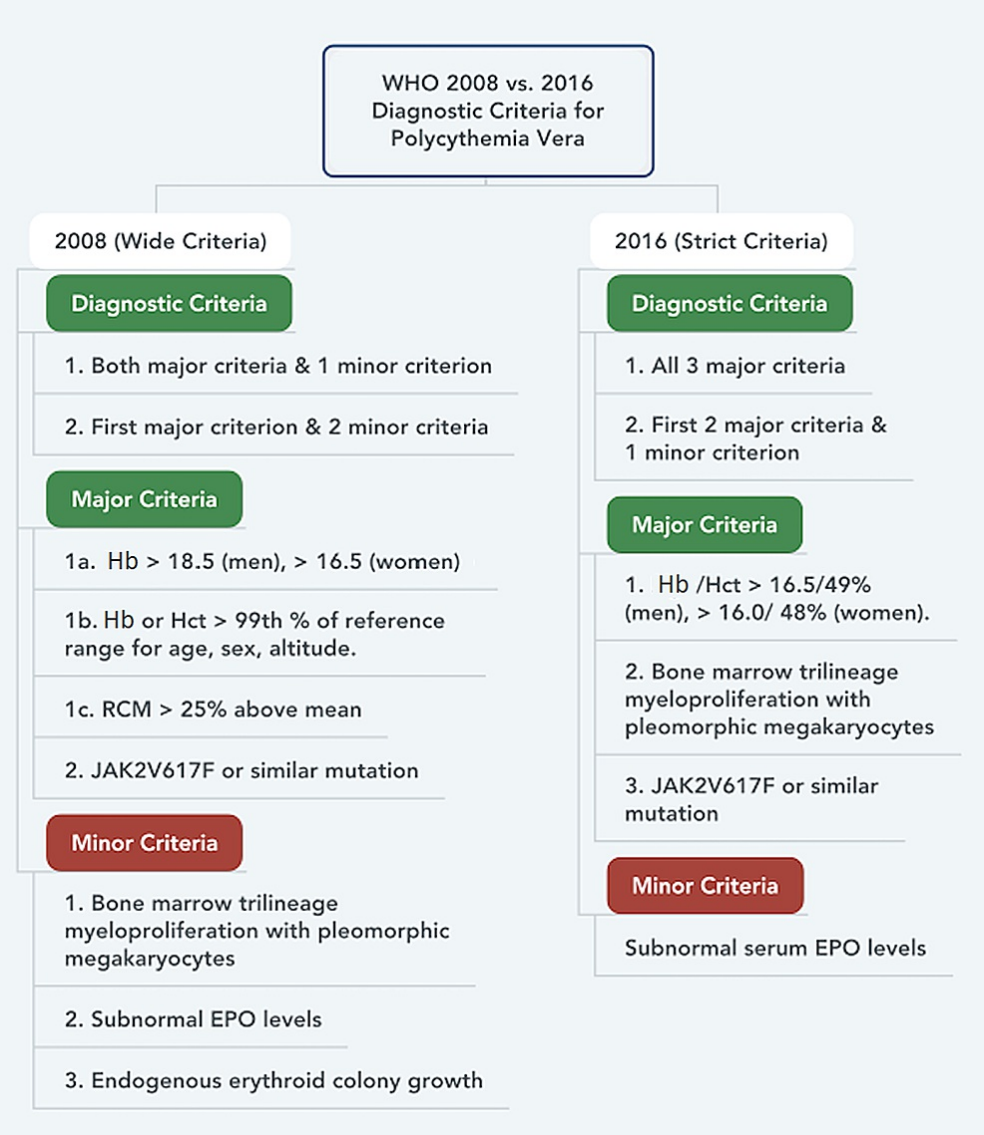
WHO diagnostic criteria for polycythemia vera (2008 vs. 2016) RCM: red cell mass; EPO: erythropoietin; Hb: hemoglobin; Hct: hematocrit

The significance for reaching the correct diagnosis is to resolve the underlying pathology that is causing erythrocytosis, and by extension its potential sequelae and to be assured that an incidence of polycythemia vera is not being missed. This led the World Health Organization (WHO) and the British Committee for Standards of Hematology (BCSH) to adopt new guidelines to avoid further underdiagnosis. In 2016, the revised diagnostic criteria for polycythemia vera were introduced and the hemoglobin and hematocrit cutoffs were among the many changes (Figure 2) [4].

The Hb levels were reduced to 16.5 g/dL from 18.5 g/dL in males and 16.0 g/dL from 16.5 g/dL in females. It also introduced a hematocrit cutoff of 48.0% for males and 49.0% for females, regardless of normal hemoglobin values. The hematocrit levels were mainly added to differentiate between essential thrombocytosis and polycythemia vera. Introducing these changes was done in response to an increase in the number of patients who were underdiagnosed because they did not meet the 2008 WHO criteria cutoff. A study reported that approximately 550,000 Americans are with undiagnosed myeloproliferative neoplasm (MPN). By using the revised criteria according to Hasselbalch, the incidence of potential PV patients has increased by 12 folds in males and three folds in females [5].

PV is notorious for its high risk of bleeding and thrombotic events. There is a growing interest in secondary erythrocytosis since the criteria update, particularly since these patients may also face similar complications. Despite efforts to clearly distinguish the various forms of erythrocytosis, there are few studies comparing secondary erythrocytosis (SE) and PV populations [6].

A total of 108,521 participants from the Danish general population were studied and results revealed that high hematocrit (women/men with hematocrit >45%/>48%) was associated with a 1.5-fold risk for myocardial infarction [7]. Interestingly, when excluding individuals with myeloproliferative neoplasms from the main analyses, results on the risk of thrombosis were similar. During a median follow-up of eight years (range: 0-13 years), 2198 individuals were diagnosed with cerebrovascular accident (CVA) and 2200 with myocardial infarction (MI) [1]. According to another study, there was an increased risk of one-year mortality in ST-elevated myocardial infarction (STEMI) patients undergoing primary percutaneous coronary intervention (PPCI) with erythrocytosis. It also showed that each 1 g/dL increment of Hb was associated with an increased risk of one-year mortality when compared to patients with normal Hb as well as patients with anemia [7]. A third study also showed that both PV and SE had similar rates of arterial or venous thrombosis at/prior to diagnosis. However, it also showed decreased thrombotic events after diagnosis of SE when compared to PV [6].

Relative erythrocytosis and absolute erythrocytosis are the two main types of erythrocytosis that have been categorized historically. Relative erythrocytosis is defined as a rise in the number of red blood cells (RBCs) per unit of blood volume. These individuals usually have a low plasma volume as a result of reduced intake or increased output, which could be due to gastrointestinal losses or genitourinary causes. Absolute erythrocytosis, on the other hand, is due to a rise in the number of erythrocytes in the blood and that can be divided into primary and secondary causes. A common cause of primary erythrocytosis is polycythemia vera (PV) which is due to a mutation in the JAK2 kinase gene. Secondary erythrocytosis, on the other hand, is defined as having normal or high levels of erythropoietin while being in a polycythemic state. Secondary polycythemia is further delineated into appropriate (physiologic) and inappropriate (pathologic) erythropoietin production [8].

When evaluating a patient with erythrocytosis, it is important to remember that almost every aspect of the history can guide the physician to narrow down the diagnosis. Patient history and symptomatology can help eliminate certain underlying differentials such as a history of chronic obstructive pulmonary disease, obstructive sleep apnea, chronic kidney disease, hyperthyroidism, smoking, and testosterone use. Only 5% of heavy chronic smokers have erythrocytosis. Erythrocytosis is commonly seen in patients with testosterone use. However, the risk of erythrocytosis in patients using testosterone pellets versus patients on intramuscular (IM) testosterone undecanoate is 35.1% and 7%, respectively [9,10].

**Figure 3:**
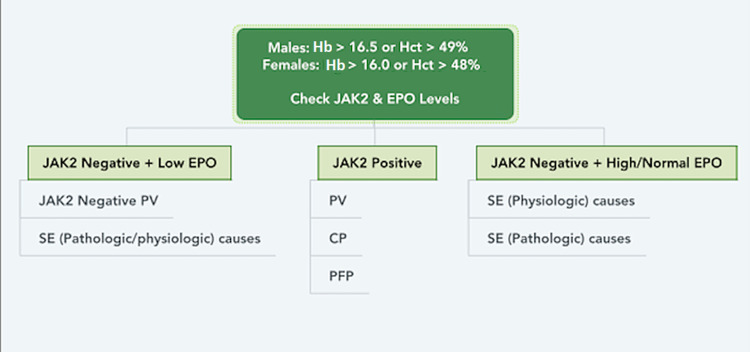
Clinical approach for the diagnosis of erythrocytosis SE: secondary erythrocytosis; PV: polycythemia vera; PFP: primary familial polycythemia; CP: congenital polycythemia; EPO: erythropoietin; Hb: hemoglobin; Hct: hematocrit

The diagnostic workup should start by confirming the erythrocytosis (Figure 3). The next step is to check JAK2 and erythropoietin (EPO) levels. This will help differentiate between primary versus secondary causes. A JAK2 kinase positive mutation confirms the presence of a myeloproliferative neoplasm (MPN) such as PV and in this case, a referral to hematology and oncology would be needed. Some cases of PV are JAK2 mutation-negative and this is where the EPO levels will be of utility. A low EPO level would need a further diagnostic workup for JAK2 negative PV, whereas a high or normal EPO level would indicate secondary causes of erythrocytosis, in which case treating the underlying cause would be the best next step in management [9].

Case 1

This patient had a positive Hb by wide criteria and a subsequent negative workup for MPNs. His EPO level was 7.3 mIU/mL, which is within the normal physiological range; however, it is considered functionally elevated given that he presently has erythrocytosis. These findings are consistent with SE based on the diagnostic workup. This can be due to chronic tobacco use, obstructive sleep apnea, or a possible paraneoplastic cause such as renal cell carcinoma. Renal cell carcinoma is less likely despite his history of tobacco use given his urinalysis being absent of RBC and lack of constitutional symptoms. Further management of this patient should include counseling on tobacco use cessation and polysomnography.

Case 2

This patient falls into the absolute SE due to physiologic EPO production. He was conclusively worked up and surgically treated for his tetralogy of Fallot with a subsequent repeat of labs indicating hemoglobin and hematocrit within the reference range. This patient does not need further laboratory work or close monitoring for erythrocytosis.

Case 3 

This patient met wide criteria, but not strict criteria by WHO standards. However, due to his history of hyperthyroidism, which is a known cause of erythrocytosis, he was started on methimazole, but further workup and management along the lines of treating hyperthyroidism are recommended. Ordering JAK2 and erythropoietin levels to confirm the absence of an underlying MPN and follow-up Hb/Hct levels is appropriate at this time.

## Conclusions

Erythrocytosis is a common finding that almost all physicians will encounter during their practice. It is critical to understand that this one lab abnormality although sometimes can be mildly elevated if not appropriately worked up may lead to complications that can be detrimental to the patient. Having an idea of the broad differential is critical. Some of which are commonly seen on a day-to-day basis in the outpatient setting. A thorough history and a pragmatic diagnostic workup are needed to guide one and subsequently ensure the appropriate management is provided.
